# Rethinking respiratory assessment: the emerging role of non-invasive *P*_mus_ estimation in real-time monitoring

**DOI:** 10.31744/einstein_journal/2025CE1962

**Published:** 2025-10-03

**Authors:** Ricardo Kenji Nawa, Germano Forti, Marcelo do Amaral Beraldo

**Affiliations:** 1 Nihon Kohden OrangeMed Santa Ana California United States Nihon Kohden OrangeMed, LLC, Santa Ana, California, United States.; 2 Universidade de São Paulo Faculdade de Medicina Hospital das Clínicas São Paulo SP Brazil Pulmonology Division, Hospital das Clínicas, Faculdade de Medicina, Universidade de São Paulo, São Paulo, SP, Brazil.

Dear Editor,

Monitoring patient effort or muscle pressure (*P*_mus_) during mechanical ventilation is essential to prevent diaphragm injury.^(1-3)^ Excessive inspiratory effort may cause patient self-inflicted lung injury and diaphragm overuse, whereas insufficient effort may result in rapid diaphragm atrophy.^(4)^

Both under- and over-assistance can disrupt the balance required to preserve respiratory muscle function.^(1-3,5)^ Quantifying *P*_mus_ enables clinicians to tailor ventilator support, ensuring protective lung strategies while maintaining appropriate diaphragmatic activity.^(1)^

**Figure 1:**
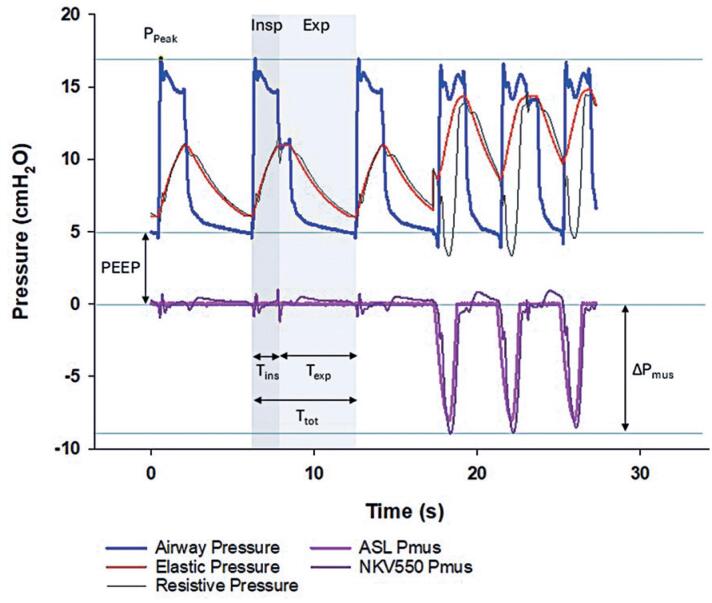
Chart representing the muscle pressure and the airway, elastic, and resistive pressures

Traditionally, patient effort has been assessed using invasive techniques, such as esophageal pressure monitoring, which require specialized equipment and clinician expertise.^(5)^ However, recent advances have enabled the development and integration of noninvasive methods for *P*_mus_ assessment in modern ventilators. These systems calculate *P*_mus_ based on the equation of motion using real-time measurements of respiratory system compliance and resistance. This approach enables accurate breath-by-breath, real-time monitoring of patient effort, offering a reliable and user-friendly alternative to invasive methods while supporting continuous bedside assessment (Figure 1).

The waveform is generated from data collected during simulated spontaneous breathing using the Active Servo Lung 5000 (ASL 5000, Software Version 3.6; IngMar Medical, Ltd., 2016; Pittsburgh, PA, United States) and NKV-550 ventilator (Nihon Kohden OrangeMed, LLC, Santa Ana, California, United States).

Exp: expiration; Insp: inspiration; PEEP: positive end-expiratory pressure; ΔP_mus_: muscle pressure; P_peak_: peak pressure; T_exp_: expiratory time; T_ins_: inspiratory time; T_tot_: total time.
